# Forest Cover Estimation in Ireland Using Radar Remote Sensing: A Comparative Analysis of Forest Cover Assessment Methodologies

**DOI:** 10.1371/journal.pone.0133583

**Published:** 2015-08-11

**Authors:** John Devaney, Brian Barrett, Frank Barrett, John Redmond, John O`Halloran

**Affiliations:** 1 School of Biological Earth and Environmental Sciences (BEES), University College Cork (UCC), Cork, Rep. of Ireland; 2 School of Geography and Archaeology, University College Cork (UCC), Cork, Rep. of Ireland; 3 Forest Service, Dept. of Agriculture, Food and the Marine, Johnstown Castle, Wexford, Rep. of Ireland; DOE Pacific Northwest National Laboratory, UNITED STATES

## Abstract

Quantification of spatial and temporal changes in forest cover is an essential component of forest monitoring programs. Due to its cloud free capability, Synthetic Aperture Radar (SAR) is an ideal source of information on forest dynamics in countries with near-constant cloud-cover. However, few studies have investigated the use of SAR for forest cover estimation in landscapes with highly sparse and fragmented forest cover. In this study, the potential use of L-band SAR for forest cover estimation in two regions (Longford and Sligo) in Ireland is investigated and compared to forest cover estimates derived from three national (Forestry2010, Prime2, National Forest Inventory), one pan-European (Forest Map 2006) and one global forest cover (Global Forest Change) product. Two machine-learning approaches (Random Forests and Extremely Randomised Trees) are evaluated. Both Random Forests and Extremely Randomised Trees classification accuracies were high (98.1–98.5%), with differences between the two classifiers being minimal (<0.5%). Increasing levels of post classification filtering led to a decrease in estimated forest area and an increase in overall accuracy of SAR-derived forest cover maps. All forest cover products were evaluated using an independent validation dataset. For the Longford region, the highest overall accuracy was recorded with the Forestry2010 dataset (97.42%) whereas in Sligo, highest overall accuracy was obtained for the Prime2 dataset (97.43%), although accuracies of SAR-derived forest maps were comparable. Our findings indicate that spaceborne radar could aid inventories in regions with low levels of forest cover in fragmented landscapes. The reduced accuracies observed for the global and pan-continental forest cover maps in comparison to national and SAR-derived forest maps indicate that caution should be exercised when applying these datasets for national reporting.

## Introduction

Globally, forests cover 31% of the total land area [1] and account for 77% of all terrestrial above ground carbon [2,3]. Loss of forest carbon through deforestation and degradation is recognised as a key driver of human-induced climate change [4,5]. Forest-related land-use changes can also have deleterious effects on biodiversity richness, water dynamics and other ecosystem services [6,7]. Hence, to enable sustainable management of forest resources, a common challenge is to quantify spatial and temporal patterns of forest cover [8–10].

Recent research has focused on large-scale mapping of forest cover and change detection on continental [11–13] and global scales [14–16], and in deforestation sensitive regions [17–19]. Data from these studies can provide valuable information on climate regulation, carbon storage and socio-economic trends [20,21]. Advances in Earth Observation (EO) technologies are likely to improve such extensive geographic estimates of forest cover and associated parameters (such as forest biomass) over the coming years [22,23]. However, accurate estimation of forest cover on a national level is also required to meet international reporting requirements and assist country specific sustainable forest management targets. For example, under the United Nations Framework Convention on Climate Change (UNFCCC) and Kyoto Protocol, Parties are required to submit annual inventories of forest land-use area and associated changes [24,25]. The monitoring of spatial and temporal changes in the distribution of forests on a local scale can also be a key component of biodiversity/ecosystem services management [26–28].

This has particular importance for Ireland, given the past widespread transformation in the areal extent of forest cover. At the beginning of the twentieth century, forest cover in Ireland was < 1% following millennia of gradual deforestation [29]. Over the last sixty years however, extensive afforestation programmes have increased forest cover to 10.5% [30], constituting one of the fastest on-going land-use changes in Europe [31]. Indeed, it is government policy in the Republic of Ireland to increase national forest cover to 18% by 2046 [32]. Driven by grant payments from the European Union, recent patterns of afforestation have shifted from large continuous blocks of state-owned forestry to small, privately-owned forest parcels [33]. This has resulted in a fragmented forest landscape in Ireland, with privately owned forests being on average < 11 ha in size [34].

A number of estimates for the extent of forest cover in the Republic of Ireland are available from both national and international sources (Table 1). These estimates have been derived using a variety of methodologies, ranging from sample based ground measurements [30], to wall-to-wall automatic classification of optical satellite imagery on a pan-European [35] and global scale [14]. While optical remote sensing has been widely used to provide spatially explicit maps of forest cover [36–38], its application is limited in countries which have near-constant cloud coverage. Due to their longer wavelengths (λ), electromagnetic waves in the microwave region of the electromagnetic spectrum are not as influenced by atmospheric conditions as sensors operating at optical wavelengths. Consequently, Synthetic Aperture Radar (SAR) provides an ideal data source for routinely tracking areas of forests for changes, irrespective of weather conditions or time of day. Most forest studies using SAR have applied to regions with dense cover and/or that are of importance in terms of the UNFCCC REDD+ (Reducing Emissions from Deforestation and Forest Degradation) mechanism [39], such as Asia [40–43], Amazonia [44–46], Africa [47], and Siberia [48,49]. In contrast, relatively few applications to areas with highly sparse and fragmented cover have been carried out.

**Table 1:** Data sources used for comparison of forest cover estimates in the Republic of Ireland, outlining the potential advantages and disadvantages of each method.

Name	Forest definition	Source/Method	Spatial resolution	Temporal resolution	Advantages	Disadvantages
Forestry2010	Minimum area of 0.1ha, trees > 5m in height and canopy cover ≥20% (or with potential to reach those limits	Irish Forest Service; automatic classification and on-screen interpretation of Landsat TM imagery (1993–1997), aerial photograph interpretation, records of state and private afforestation and historic forest maps	<10m	Periodic (Non-uniform)	High spatial resolution, all newly grant-aided afforested areas accurately captured	Deforestation areas may not be accurately reported, no account of successional forests (e.g. scrub woodland encroachment on abandoned peat)
National Forest Inventory (NFI)	Minimum area of 0.1ha, trees > 5m in height and canopy cover ≥20% (or with potential to reach those limits	Irish Forest Service; 1827 500m2 forest survey plots and aerial photointerpretation of land-use of 17,423 grid points	<10m	6 years	Fully ground truthed survey plots	Sample-based, not spatially explicit, wide confidence limits on deforestation estimations
Prime2	Not specified	Ordinance Survey Ireland (OSi): Digitisation of aerial photographs, OSi databases, boundaries datasets	<10m	Not specified	High spatial resolution, includes some areas not captured in the Forestry2010 dataset (e.g. newly developed scrub forest)	No systematic update cycle defined. Some reported errors in interpretation and classification (e.g. miscanthus grass bioenergy crops misclassified as forest)
JRC Forest Map 2006	Areas occupied by forest with native or exotic coniferous and/or deciduous trees and which can be used for the production of timber or other forest products. Forest trees are under normal climatic conditions higher than 5 m with a canopy closure of 30%	Joint Research Centre, Italy: Supervised classificiation of optical remote sensing data (Landsat ETM+, IRS LISS-III, Spot 4–5, MODIS)	25m	Periodic (Non-uniform)	Pan-European coverage	Low accuracies evident for Ireland—large underrepresentation of forest cover
RADAR Imagery		ESA/Jaxa: ALOS PALSAR Fine Beam Dual (FBD) polarisation	15m	46 days	High spatial and temporal resolution	Lack of national expertise, national coverage can be expensive
Global Forest Change 2000–2012	Percentage cover of vegetation >5m mapped. For this study, areas of tree cover >20% considered forest	University of Maryland, USA/Google Earth Engine; Time-series analysis of 654,178 Landsat ETM+ images, classified using a decision tree classifier	30m	Annual*	Freely available global data with planned annual updates	Forest area vary widely with national forest statistics, differences in forest definitions

* Annual updates have been proposed by Hansen et al. (2013).

The most common spaceborne microwave remote sensing instruments used for forest applications operate at X- (λ~3cm), C- (λ~5cm) and L-band (λ~24cm). The shorter wavelength radar (X-band) interacts mainly with the tops of the canopy cover while longer wavelengths (L-band) are able to penetrate further into the canopy. L-band undergoes multiple scattering between the canopy, trunks and soil and is therefore significantly more useful in forest and vegetation studies as it is able to penetrate deeper into the canopy cover [50]. However, despite these advantages, the use of radar is not yet an established method for forest monitoring. This can be attributed to several factors: the limited availability of long-wavelength data, moisture (soil and vegetation) influences on the backscatter signal, continuity of data provision, and the complexity of interpreting the data [40,51]. Nonetheless, various studies have been carried out that have confirmed the potential of these frequencies for forest observations [52–56]. The almost five years of continuous data from the Japanese Aerospace Exploration Agency (JAXA) Advanced Land Observing Satellite (ALOS) mission [57] provides an unprecedented archive of multi-polarisation L-band data that can be used to evaluate and optimise methods for exploiting long-wavelength radar data for forest monitoring from future missions.

In this study, we investigate the potential use of L-band SAR for forest cover monitoring in fragmented forest landscapes. There are two specific objectives of the study. Firstly, the use of L-band SAR data for forest cover mapping in two study areas in Ireland is examined. Secondly, using independent accuracy assessment, a comparative analysis is carried out on radar-based forest cover estimates and three national, one pan-European and one global forest cover product. For the purposes of this analysis, the forest definition used for international forest reporting in Ireland (land with a minimum area of 0.1ha, trees > 5m in height and canopy cover ≥20%) has been adopted. The results of this study will help inform future forest extent estimation and monitoring approaches in Ireland.

## Materials and Methods

### 2.1 Study areas

**Fig 1:**
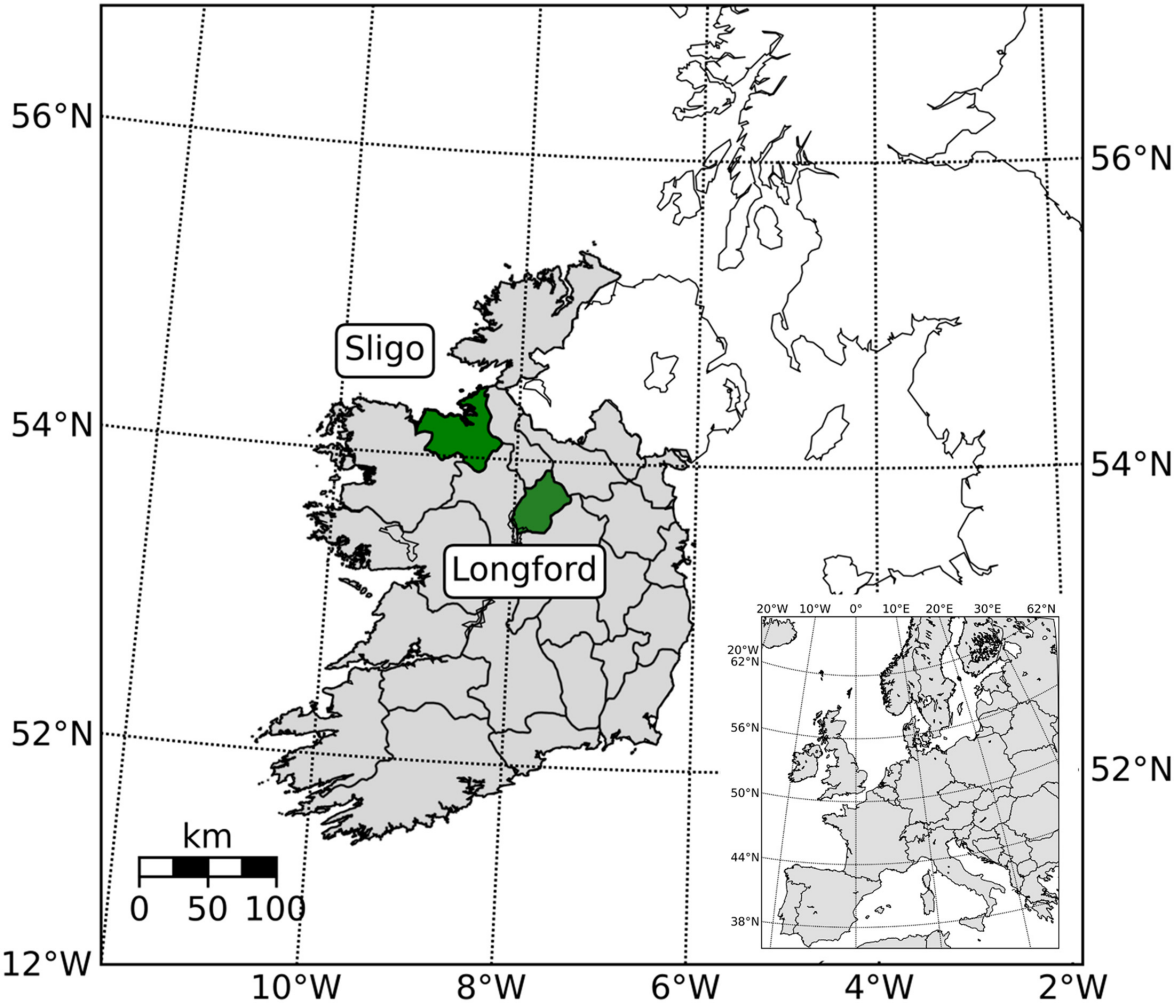
Location of counties Longford and Sligo in the Republic of Ireland (shaded green).

Two distinct study areas were selected to investigate potential geographic variation in the accuracy of forest cover estimation methodologies (see Fig 1). The study areas encompassed two Irish counties, Longford and Sligo, which, based on results from Ireland’s second National Forest Inventory (NFI) in 2012, have 7.7% and 11.2% forest cover respectively (as a proportion of total land area) [30]. The two areas vary considerably with respect to the nature of forest cover. 71.4% of forests in Longford are in private ownership. Large areas of state-owned non-native conifer dominated forests make up a considerable proportion of forest cover in Sligo. In contrast, state-owned forests constitute 48.1% of the forest area in Sligo. Sitka spruce (*Picea sitchensis*) is the main forest tree species in Sligo accounting for 59.3% of the stocked forest area, with native forest tree species accounting for only 24%. Although the non-native conifer species Sitka spruce and Norway spruce (*Picea abies*) are also the most common forest tree species in Longford (19.4% and 34.3% of the stocked forest area respectively), native species (principally ash *Fraxinus excelsior*, birch *Betula pubescens* and alder *Alnus glutinosa*) are more common (36.6% of the forest area) [30]. Both counties are characterised by differences in climate and topography. Longford is 1091km^2^ in size and is predominantly lowland, with the northwest of the county primarily consisting of drumlin topography. The Sligo region has an area of 1837km^2^ and has two distinctive upland regions, the Dartry Mountains (highest altitude of 647m) to the north of the county and the Ox Mountains (highest altitude of 544m) to the south west.

### 2.2 Datasets

#### 2.2.1 ALOS-PALSAR

The ALOS satellite was launched on 19^th^ January 2006 and operated until 12^th^ May 2011 [57]. The Phased Array-type L-band Synthetic Aperture Radar (PALSAR) instrument on board ALOS operated at L-band (λ~24cm) and provided fully polarimetric capabilities. Table 2 displays the data characteristics for each acquisition for both study sites. All acquisitions were acquired from ascending orbits in Fine Beam Dual-polarisation (FBD) mode (comprising both horizontal transmit and horizontal receive (HH) and horizontal transmit and vertical receive (HV) data) and have a swath width of 70km. Each image has an off-nadir angle of 34.3° (corresponding to an incidence angle at scene centre of approximately 38°). Two frames (acquired on the same acquisition date) were required to obtain complete coverage for each study area. The satellite had a repeat-pass cycle of 46 days and operated a systematic observation strategy, whereby the sensor mode, geographical region and acquisition timing were fixed for the duration of the mission. The primary benefit of such a strategy was that systematic and regionally consistent data observations over all land surfaces could be provided. FBD mode acquisitions were scheduled during the northern hemisphere summer months with a minimum of two acquisitions (during consecutive 46-day cycles) per year. For this study, acquisitions from June 2010 were used.

**Table 2:** PALSAR Data Characteristics.

Date	Sensor	Mode	*λ* (cm)	Polarisation	*θ*	Orbit	Track	Frame
**Sligo**								
2010-06-07	PALSAR	FBD	23.6	HH/HV	~38°	23279	3	1070
2010-06-07	PALSAR	FBD	23.6	HH/HV	~38°	23279	3	1080
**Longford**								
2010-06-19	PALSAR	FBD	23.6	HH/HV	~38°	23454	1	1060
2010-06-19	PALSAR	FBD	23.6	HH/HV	~38°	23454	1	1070

λ = wavelength

θ = incidence angle

#### 2.2.2 Forestry2010

Since 1995, the Irish Forest Service have produced spatial datasets detailing the extent of the forest estate in the Republic of Ireland [58]. Initially known as the Forest Inventory and Planning System (FIPS), this spatial dataset was derived from automatic classification and on-screen interpretation of Landsat TM imagery (1993–1997), panchromatic orthophotos (1995), and Ordnance Survey Ireland (OSi) 25-inch map series [58]. Forest boundaries were digitised to within 2m accuracy of the orthophotos and the OSi 25-inch map series. In 1998, records of private afforestation since 1980, based on digitisation of hardcopy maps and OSi orthophoto interpretation were appended to the FIPS dataset. Since then, all newly afforested areas in receipt of grant payments have been added to this dataset on an annual basis. For the purposes of this study, all post 2010 afforestation areas were removed from the current dataset, along with any non-forest land cover areas, to create Forestry2010.

#### 2.2.3 Prime2

Prime2, released in late 2014, is an object-based spatial data storage model, created by OSi. It provides a highly detailed database of all topological features on the landscape and is derived from digitisation of OSi orthophotography and existing boundaries datasets. All objects in Prime2 are classified by *form* and *function* attributes, which loosely represent their real-world cover and use. There are five separate but complementary layers that together make up the seamless Prime2: i) Way (e.g. roads, railway tracks), ii) Water (e.g. fresh & salt water bodies), iii) Vegetation (e.g. fields, forests), iv) Exposed (e.g. sand, rock outcrops), and v) Artificial (e.g. concrete areas). For this study, only the Vegetation layer was of interest and all forest classes (Woodland Coniferous, Woodland Deciduous, and Woodland Mixed) were extracted to create the Prime2 forest cover map.

#### 2.2.4 Irish National Forest Inventory

The second Irish NFI began in 2009 and was completed in 2012 [30]. The NFI is a detailed survey of permanent forest sample plots with the purpose of recording and assessing the extent and nature of Ireland’s forests in an accurate and repeatable manner. Based on a national, randomised systematic 2 x 2 km grid sample design, aerial photo interpretation of 17,423 grid points was carried out in 2006 and again in 2012. Following ground checks, all potential forests plots meeting the national forest definition were established as permanent sample plots. This is a modified approach of one of the most commonly used NFI methodologies worldwide [59]. For the second NFI, 1827 permanent sample plots were established. Based on the sampling design used, each forest plot (500 m^2^) is representative of 400 ha of forest nationally. A suite of ground measurements are recorded at each forest plot. Estimations of NFI forest cover estimates in the study areas were taken from the results of the second NFI, for which field surveys were carried out in 2010–2012 [30]. All area estimates are taken from the “Stocked Forest Area” statistics of the NFI.

#### 2.2.5 Forest Map 2006

The Forest Map 2006 is a pan-European forest cover map, produced using a multilayer perceptron Artificial Neural Network (ANN) applied to optical remote sensing data [60]. Wall-to-wall forest cover data for Europe and selected neighbouring countries is available at 25 x 25m pixel size using images acquired during 2005–2007 from the medium resolution Linear Imaging Self-Scanner (LISS-3) sensor on board the Indian Remote Sensing satellite (IRS-P6), Satellite Pour l’Observation de la Terre (SPOT 4/5) and Moderate Resolution Imaging Spectroradiometer (MODIS) imagery. The CORINE Land Cover (CLC) Map 2006 was used as training data and the EU Land Use and land Cover Area Statistical survey (LUCAS) data and eForest Platform (harmonised NFI data from all EU-27 member states) used for its validation. The Forest Map 2006 data covering Longford and Sligo was acquired from the EU Joint Research Centre (JRC) at http://forest.jrc.ec.europa.eu/download/data/forest-data-download.

#### 2.2.6 Global Forest Change 2000–2012

In 2013, the findings of a major study on the use of EO satellite data to map global forest loss and gain from 2000–2012 were published by Hansen *et*. *al*. [14]. A time-series analysis of 654,178 Landsat Enhanced Thematic Mapper plus (ETM+) images were classified using a decision tree classifier [61] to characterise global forest cover and change during the period 2000 to 2012 at a resolution of ~30m. This data analysis was performed using Google Earth Engine and is available on-line from: http://earthenginepartners.appspot.com/science-2013-global-forest. Detailed information on how this dataset was derived is available in the supplementary material of Hansen *et*. *al*. [14]. For this study, a subset of the global forest cover datasets covering the study areas was extracted. The data was re-classified where forest canopy cover ≥20% equalled forest and canopy cover < 20% equalled non-forest cover. The global forest cover loss and gain between the period 2000 and 2012 was added to the 2000 global forest canopy cover product to create a forest cover 2012 product. Finally, forest cover loss during the years 2011 and 2012 was removed to produce a 2010 forest cover dataset for comparison with the other forest cover datasets.

### 2.3 SAR Pre-processing

All data were delivered as single look complex (SLC) data products from the European Space Agency (ESA) (Cat-1 ID 14194). Multi-looking factors of 1 (in range) and 4 (in azimuth) for the PALSAR FBD data were applied to create 15 x 15m pixels. The data scenes were subsequently speckle filtered using a de Grandi multi-temporal speckle filter and radiometrically and geometrically calibrated and converted to decibel (dB), according to the formula by [57]:

$$\gamma_{\left(dB\right)}^{0}\;=\;10_{\text{log}10}DN^{2}+cf$$ (1)

where γ° is the backscattering coefficient (dB), *DN* is the pixel digital number value in HH or HV, and *cf* is the absolute correction factor of -83.

**Fig 2:**
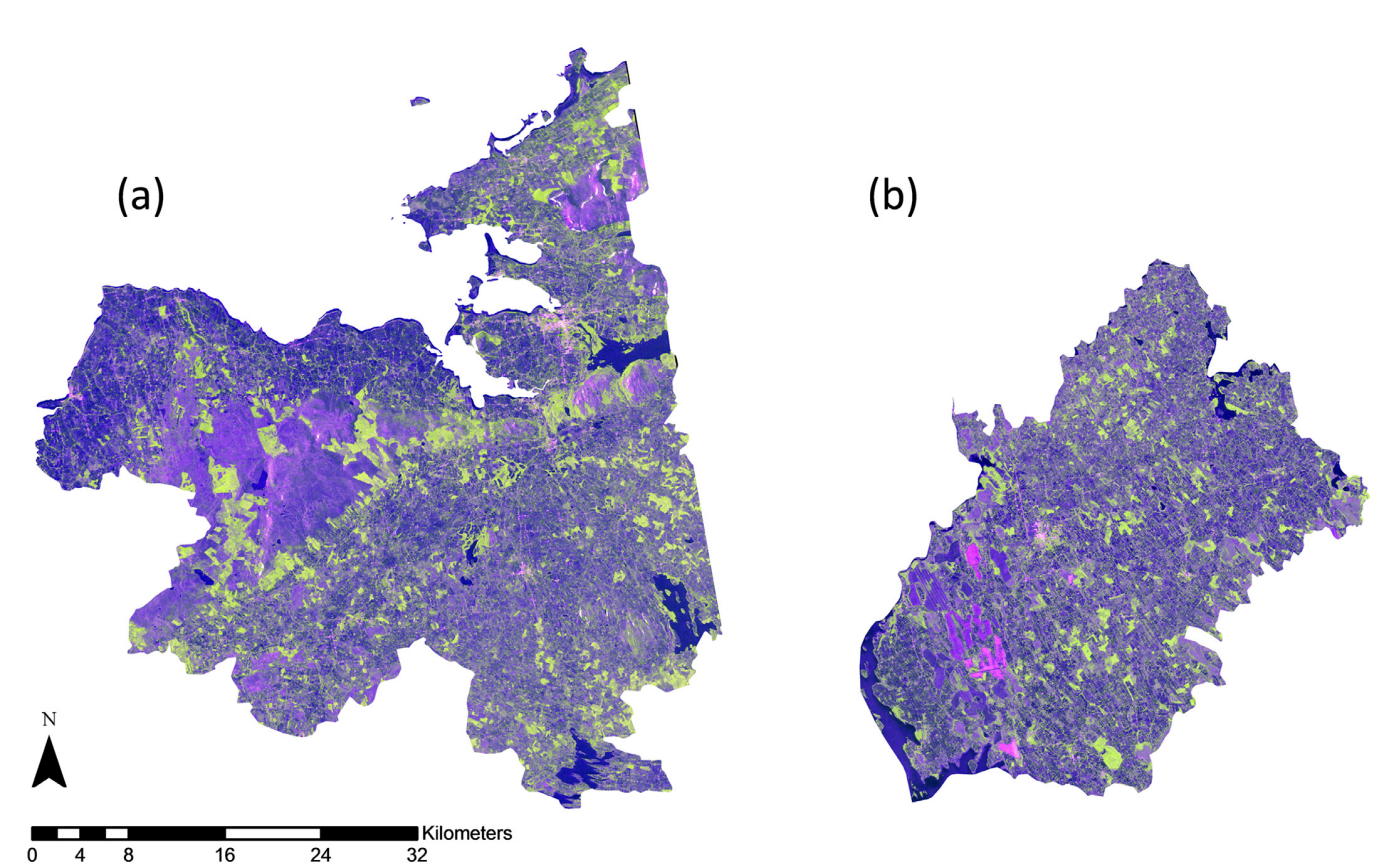
PALSAR false colour composites (HH backscatter (red)–HV backscatter (green)–HH/HV backscatter ratio (blue)) for Sligo (a), and Longford (b). (Source: European Space Agency)

An OSi 10m spatial resolution DEM with a vertical accuracy of 0.5m and several ground control points (GCPs) were used to geometrically correct the SAR scenes to the Irish Transverse Mercator (ITM) projection using a Range-Doppler approach. No terrain distortions were present in the Longford dataset as a result of its low-lying topography. Conversely, the scenes for Sligo needed to be masked for certain terrain-induced distortions (e.g. layover and shadowing) due to a more varying topography. Approximately 0.2% (2.73km^2^) of the total county area (1837km^2^) was subsequently masked out. The areas of layover and shadow were calculated using the local incidence angle (angle between the normal to the backscattering object and the incoming radar signal) as generated using the DEM, where negative values corresponded to layover areas and values higher than 90° corresponded to shadow areas. In a final step, the two scenes covering each area were mosaiced. All SAR processing was carried out using SARscape 5.0.001 software within an ENVI 4.8 environment. Fig 2 displays a false colour composite mosaic for both counties. Forest areas appear in green, water bodies appear in blue, and agricultural fields and peatlands appear in varying shades of purple. Sligo and Longford town, which have almost 20,000 and 8,000 inhabitants respectively, appear in light shades of pink.

### 2.4 Training data

A 15 x 15m grid overlaid on the radar datasets was used to extract γ° values from the cell centroid locations. A total of 2378 training samples were selected for Longford (forest = 1189; non-forest = 1189), and 7890 training samples for Sligo (forest = 3945; non-forest = 3945). The location of training samples is provided in S1 and S2 Figs These samples were selected on the basis of reference information obtained from ancillary datasets including OSi aerial imagery and Bing Maps image layer within ArcGIS 10 (ESRI Inc.) to provide spatially representative samples for both classes. The validation followed the widely used *k*-fold cross validation approach [62]. In this approach, the training samples are separated equally into a user-defined number of subsets, *k*. For this study, *k* was set to 5. During the training stage, one of the *k* subsets is chosen for validation and the remaining (*k*-1) subsets for training. This process is repeated sequentially until all the data sets have been tested. The classification errors computed from the validation sets are then averaged over the *k*-trials. The *k*-fold cross validation accuracy is the percentage of validation samples that are correctly classified, and this measure provides a more objective and accurate estimate of classification accuracy than the traditionally used 1-fold method [63]. A further advantage is that all the samples are eventually used for both training and validating the classifiers, thereby minimising the impact of the training data selection.

### 2.5 Classifiers

Several methods for forest cover classification can be found in the literature (e.g. [64–66]). In this study, two machine-learning algorithms; Random Forests (RF) [67] and Extremely Randomised Trees (ERT) [68] were applied to the radar backscatter (HH, HV, and HV/HH ratio) and ancillary (elevation, slope, soils and subsoils) datasets to distinguish between forest and non-forest areas on a per-pixel basis. To assess the influence of the ancillary variables on the classification accuracies, the variable importance ranking derived from the RF algorithm was analysed to evaluate the contribution of each variable to the classification accuracies. As recommended by Díaz-Uriarte and De Andres [69], a RF model with 5000 trees was used to calculate the variable contributions.

Machine Learning techniques such as RF have been gaining more widespread use in forest cover and carbon mapping applications (e.g. [44,70,71]) as they can accept a variety of measurement scales for both numeric and categorical variables, handle many input variables, do not rely on the data distribution and do not suffer from overfitting [72]. Both classifiers were implemented in Python v2.7.6 using the open-source Scikit-learn module [73].

#### 2.5.1 Random Forests

Random Forests (RF) builds an ensemble of individual decision-tree classifiers that are later combined using a majority voting scheme to improve predictive accuracy. The individual trees are constructed using a bootstrap sample of the training data, whereby the training is performed on two thirds of the data samples and the remaining one third of the data samples are omitted. These ‘out-of-bag’ (OOB) samples are used to test the classification during each iteration and estimate the OOB error. For this study, the RF algorithm was run using a 200-tree ensemble and a random sample of predictor variables at each node equal to the square root of the total number of predictor variables. RF can also provide the relative importance of each of the variables used in the model formulation which can provide valuable insight into the contribution of each variable to the classification accuracy (see S1, S2 and S3 Tables).

#### 2.5.2 Extremely Randomised Trees

Extremely Randomised Trees (ERT) or Extra-Trees is a relatively underused (in EO applications) tree-based ensemble classifier, introduced by Guerts *et al*. [68], that has been shown to be effective in high-dimensional classification problems, predominantly in biomedical imaging (e.g. [74–77]). Few applications of ERT to multi-configuration SAR exist [78]. The primary differences between ERT and RF is that ERT randomly chooses the node split when constructing each tree (as opposed to searching for the optimal split node) and uses the same input training data to train all individual trees. Increasing the randomisation reduces the variance among trees, while using the full training dataset rather than bootstrap samples minimises the bias. Similar to RF, the number of the trees was set to 200 and the number of variables to split at each node was set to the square root of the number of predictor variables.

#### 2.5.3 Post-classification filtering

Images obtained from coherent sensors such as SARs are characterised by speckle. Multi-looking and speckle filtering cannot remove entirely the presence of speckle from the backscatter images and this subsequently impacts on the output classification maps. In addition, small groups of trees and hedgerows that were classified as forest needed to be removed as they do not meet the forest definition used in this study. For both the RF and ERT derived forest maps, a series of post-classification filtering procedures were applied (ranging on a scale from 1 (weak filtering) to 5 (strong filtering)) in order to compare overall accuracies and determine an optimal post-classification filtering procedure. The connectivity of all originally classified forest pixels was analysed and this informed the filtering process. For scale 1, all contiguous pixel regions of 7 or less were dissolved and all pixel regions of 71 or less were dissolved for scale 5.

### 2.6 Data harmonisation

The extent of forest cover in two regions in Ireland was evaluated using ALOS PALSAR imagery from 2010 and compared with estimates from three national sources (Forestry2010, Prime2, NFI), one pan-European (JRC Forest Map 2006) and one global forest map (Global Forest Change 2000–2012). Spatially harmonising the different datasets before comparison is critical [79]. The JRC2006 and Global Forest cover products were re-projected into the Irish Transverse Mercator (ITM) projection using a bilinear resampling. Raster maps were converted to polygon shapefiles with simplified polygons. For all maps, forest polygons were dissolved to derive a discrete polygon for each continuous forest parcel and the area (ha) of each forest parcel was calculated. Any resulting polygons <0.1 ha were removed as they fail to meet the forest definition. As radar and Prime2 coverage were not complete for both Longford and Sligo, all maps were clipped to a vector boundary layer of areas common to all datasets. Each forest map was also used to populate the OSi Prime2 object-based database using a simple majority rule. From each map, total forest area and average forest size was calculated for both counties. It is noted that although SAR-derived maps, GFC, JRC and NFI stocked forest areas provide estimates of forest *land-cover*, Forestry2010 and Prime2 are inherently forest *land-use* maps. Despite the modification of the Forestry2010 to exclude non-forest land-use areas and the exclusion of forest roads from Prime2, these datasets will include recently clear-felled areas without tree cover and open spaces within the forest.

### 2.7 Accuracy assessment of forest datasets

An independent accuracy assessment was carried out on all forest cover maps. Forest/non-forest ground survey point data were combined from a number of available sources. NFI forest plots, LUCAS 2009 and the national survey of native woodlands in Ireland survey plots were combined to create an independent accuracy assessment dataset. Ground visits to all NFI permanent sample forest plots in Longford and Sligo were carried out during 2010–2012. LUCAS (Land Use/Cover Area frame Survey) is a ground-based survey, with direct observations of land use/cover recorded by field surveyors at 270,000 permanent sampling units throughout the European Union in 2009. It is managed by Eurostat (Directorate-General of the European Commission acting as a statistical office) and carried out every three years [80]. The national survey of native woodlands in Ireland was a comprehensive ground survey of native woodlands in Ireland [81]. A total of 1,320 forest plots were surveyed in the Republic of Ireland during the period 2003–2007. Ground survey forest/non forest points from Longford and Sligo were combined from these sources to create an independent accuracy assessment dataset. For Longford, the dataset contained 77 forest and 273 non-forest samples, and 90 forest and 455 non-forest samples for Sligo. The overall accuracies of forest/non-forest cover maps were calculated using this independent accuracy assessment dataset.

## Results

### 3.1 Backscatter analysis

**Fig 3:**
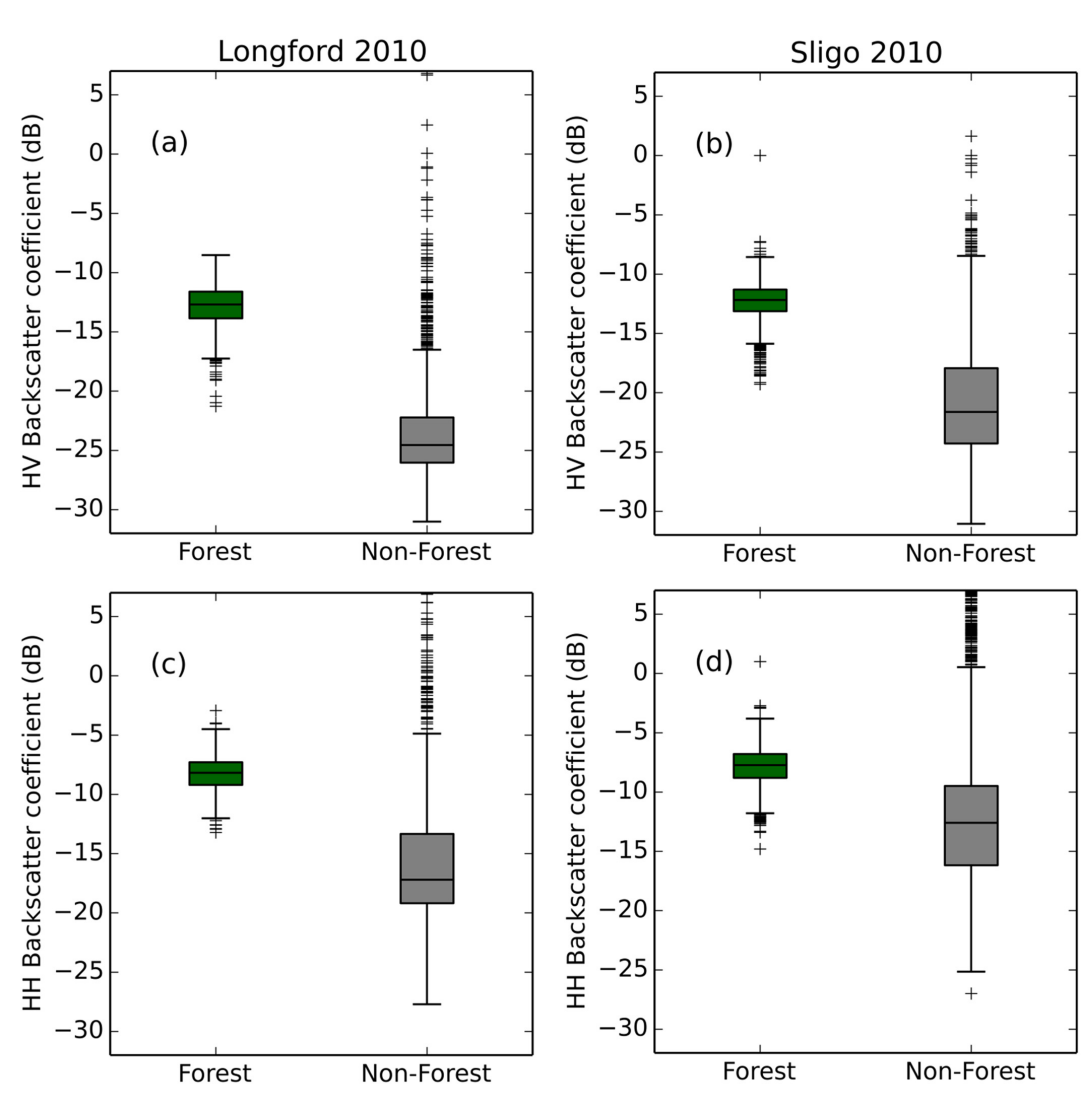
Box plots of median L-band HH and HV backscatter (γ°) for forest and non-forest training samples for Longford and Sligo.

The distribution of radar backscatter (γ°) values for the forest and non-forest classes in both study areas is shown in Fig 3. The forest backscatter samples have a high level of agreement with each other. As expected, the non-forest samples display a much wider range as they include samples from multiple cover types (e.g. settlement, water, grassland, peatland). Nonetheless, significant differences (p <0.001) in the mean γ° for both HV and HH polarisations are obtained. Sligo has slightly higher mean γ° values in both HH (-7.8 ± 1.7dB) and HV (-12.2 ± 1.8dB) polarisation, compared to Longford (-8.3 ± 1.4dB and -12.9 ± 1.7dB). The average difference between forest and non-forest is 7.5dB and 10 dB for Longford and 4.5dB and 8.6dB for Sligo in both HH and HV polarisations, respectively. At L-band, the backscatter response is a mixture of backscattering from branches, trunks, and trunk-ground backscattering. Typically, trees with higher biomass result in a stronger backscatter signal being recorded by the sensor. The areas with the strongest response in the cross-polarised images are indicative of signal depolarisation, caused by volume scattering due to dense vegetation cover or multiple scattering due to significant surface roughness. Surface scattering does not cause significant depolarisation and therefore cross-polarised images can generally be used to discriminate between these different scattering mechanisms.

### 3.2 SAR Classification accuracies

The RF and ERT radar classification results for Longford and Sligo are summarised in Table 3. Overall accuracies were high (98.1–98.5%), with differences between the two classifiers being minimal (<0.5%) and consistent results for both study areas with associated high kappa coefficients (κ = 0.96–0.97). User`s accuracy was marginally higher for the forest class than the non-forest class, suggesting a greater tendency of the algorithms to slightly overestimate forest extent (i.e. misclassify non-forest as forest). Differences between forest area estimates from the various post-classification filtering procedures for SAR derived maps are shown in Table 4. For non-Prime2 populated forest area maps, an increased level of post-classification filtering resulted in a reduction in the overall estimated forest area, an increase in the mean forest size, and an increase in overall accuracy following independent accuracy assessment (1.5%- 5.5%) in all cases. For example, in Longford, total estimated forest area decreased from 15,432.8 ha to 11,848.8 ha between the ERT-1 and ERT-5 filtering levels, with a corresponding mean forest size increase from 1.51 ha to 12.51 ha. Similarly, forest area decreased from 22,038.0 ha to 19,533.9 ha between ERT-1 and ERT-5 for Sligo. In general, this reduction in area estimation was associated with an increase in overall accuracy of forest cover maps. For non-Prime2 populated maps, with the exception of the Sligo RF classifier, highest accuracies were recorded at the strongest filtering level. Overall, higher accuracies were reported when using the RF classifier in comparison to ERT for both counties. However, this trend was reversed when forest cover maps were populated into Prime2, with higher accuracies recorded for ERT Prime2 populated forest cover maps for Longford and Sligo. For Longford, the highest overall accuracy was recorded with the Prime2 populated ERT-4 forest map. The highest accuracy forest map for Sligo was the Prime2 populated ERT-4 estimate.

**Table 3:** Radar classification results (RF = Random Forests, ERT = Extremely Randomised Trees, PA = Producer`s Accuracy, UA = User`s Accuracy).

	Longford				Sligo			
	RF		ERT		RF		ERT	
	PA	UA	PA	UA	PA	UA	PA	UA
Forest	0.97	0.99	0.97	0.99	0.98	0.98	0.98	0.99
Non-Forest	0.99	0.97	0.99	0.97	0.98	0.98	0.99	0.98
Overall Accuracy	98.1%		98.4%		98.2%		98.5%	
Kappa coefficient	0.96		0.97		0.96		0.97	

**Table 4:** Extremely Randomised Tress (ERT) and Random Forests (RF) forest area (ha) estimates, mean forest size (ha) and overall accuracy (based on independent accuracy assessment dataset) for each of the different post-classification levels for Longford and Sligo. Corresponding metrics are presented for the Prime2 populated datasets.

Region			Non Prime2 populated			Prime2 populated	
		Forest area (ha)	Mean Forest size (ha)	Overall accuracy (%)	Forest area (ha)	Mean Forest size (ha)	Overall accuracy (%)
Longford	ERT-1	15,432.80	1.51	90.86	9,915.30	2.21	96
	ERT-2	14,779.20	2.23	92.57	9,832.60	2.31	96
	ERT-3	13,759.40	4.03	93.43	9,695.80	2.58	96.28
	ERT-4	13,124.30	5.84	95.14	9,576.60	2.82	96.85
	ERT-5	11,848.80	12.51	96.28	9,346.60	3.64	96.28
	RF-1	13,271.50	1.44	93.71	8,945.50	2.21	95.42
	RF-2	12,652.70	2.17	94.86	8,890.90	2.36	95.42
	RF-3	11,680.40	4.13	96	8,711.50	2.73	96.28
	RF-4	11,111.90	6.16	96.28	8,568.70	3.06	96
	RF-5	10,086.70	13.35	96.28	8,184.90	3.87	96.57
Sligo	ERT-1	22,038.00	2.72	92.66	18,662.50	3.74	95.96
	ERT-2	21,547.00	4.16	93.03	18,533.30	3.95	95.96
	ERT-3	20,809.60	7.59	93.58	18,336.30	4.45	96.15
	ERT-4	20,403.40	10.6	93.95	18,231.00	4.87	96.15
	ERT-5	19,533.90	19.79	94.13	17,745.80	5.77	95.78
	RF-1	20,055.80	2.59	93.03	17,342.90	3.78	95.41
	RF-2	19,566.60	4.04	93.03	17,253.50	4.01	95.41
	RF-3	18,859.50	7.5	94.5	17,101.50	4.59	95.41
	RF-4	18,486.70	10.43	94.86	17,008.00	5	95.41
	RF-5	17,731.30	18.29	94.68	16,658.00	5.92	95.41

### 3.3 Impact of ancillary data on classification accuracy

**Fig 4:**
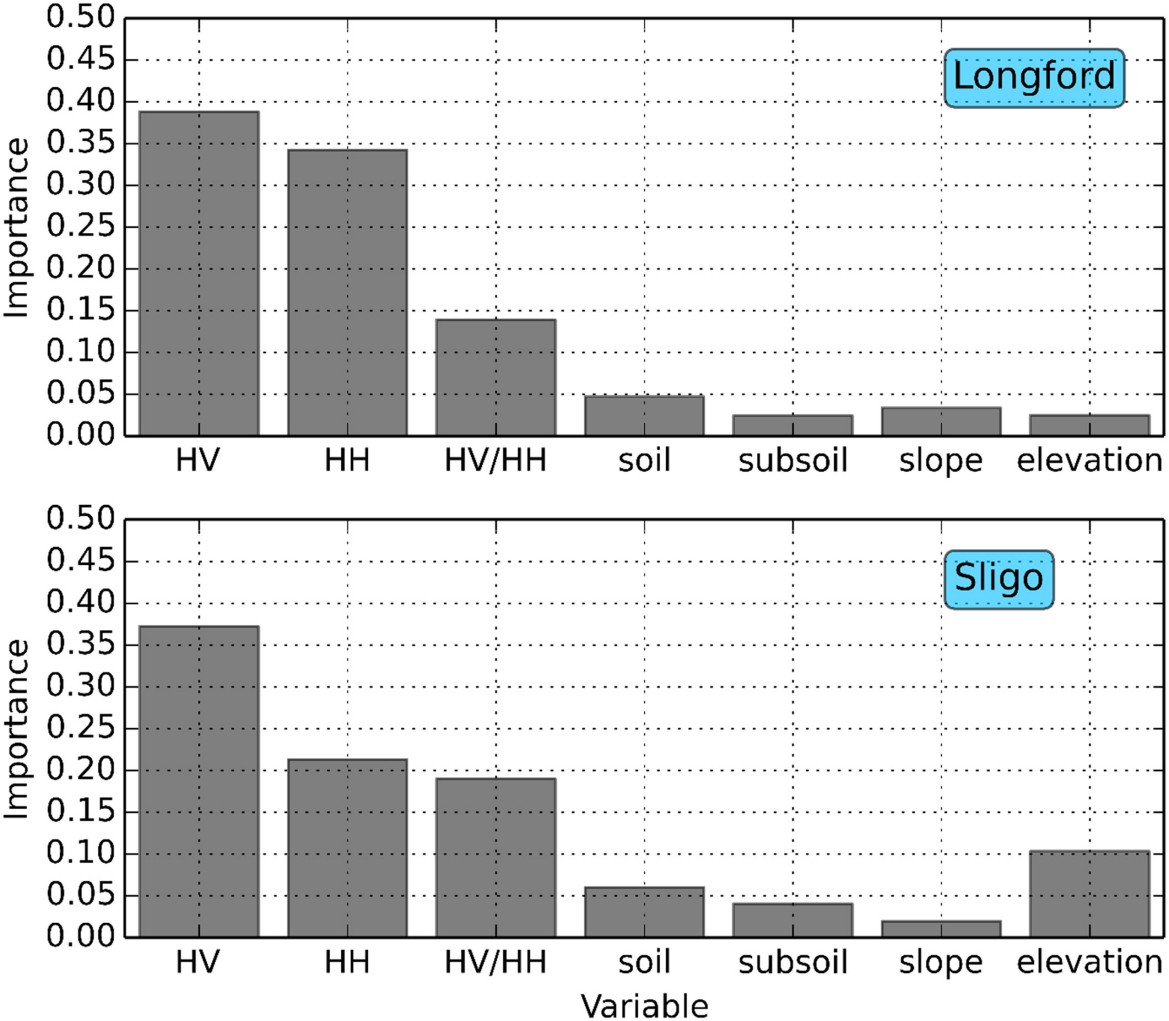
Variable importance scores of the radar backscatter intensities and ancillary data for Longford (top) and Sligo (bottom).

To assess the influence of the ancillary variables on classification accuracies, the variable importance ranking derived from the RF algorithm was analysed. As can be seen from Fig 4, the radar HV intensity data have the highest importance for both counties, followed by the HH intensity data and the HV/HH ratio data. The ancillary datasets have the lowest importance scores. The accumulation of scores for all variables equals one. Based on these scores, the ancillary variables have little contribution to the classification accuracies. To quantitatively assess this, the classifications were re-run to assess the influence of the ancillary variables on the original classification accuracies. Table 5 displays the results of the RF and ERT classifiers for (i) all variables, (ii) soil and subsoil information is excluded, (iii) slope and elevation information is excluded, and (iv) the radar intensities only. The accuracies when either the soil or elevation data are omitted are approximately the same for both classifiers in each county, with the lowest accuracies being achieved when only the radar data are considered. The increased importance score of the elevation data for Sligo may be explained by the fact that many forests in Sligo occur in upland areas and that the topographic relief of Longford is relatively flat. Overall, the inclusion of the ancillary data contributes between 2–4% to the overall accuracies when considering a forest—non-forest classification approach. Not surprisingly, these differences increase when several different land cover classes are included (as can be seen in S1, S2 and S3 Tables and see section 4.3) and this is in agreement with previous studies (e.g. [78] and [82]). The results from this study indicate that even if ancillary variables are not available/or used in a forest—non-forest classification, high accuracies can still be obtained using only the radar intensity data.

**Table 5:** Comparison of classification accuracies with and without the ancillary data.

		Longford				Sligo			
		RF		ERT		RF		ERT	
		OA	Kappa	OA	Kappa	OA	Kappa	OA	Kappa
i)	All variables	98.1%	0.96	98.4%	0.97	98.2%	0.96	98.5%	0.97
ii)	No soil	96.3%	0.93	96.5%	0.93	96.8%	0.94	96.9%	0.94
iii)	No elevation	97.8%	0.96	97.9%	0.96	96.6%	0.93	96.7%	0.93
iv)	Radar only	95.7%	0.91	95.1%	0.90	94.6%	0.89	94.0%	0.88

### 3.4 Comparison of SAR forest cover estimates with existing forest cover estimates

**Fig 5:**
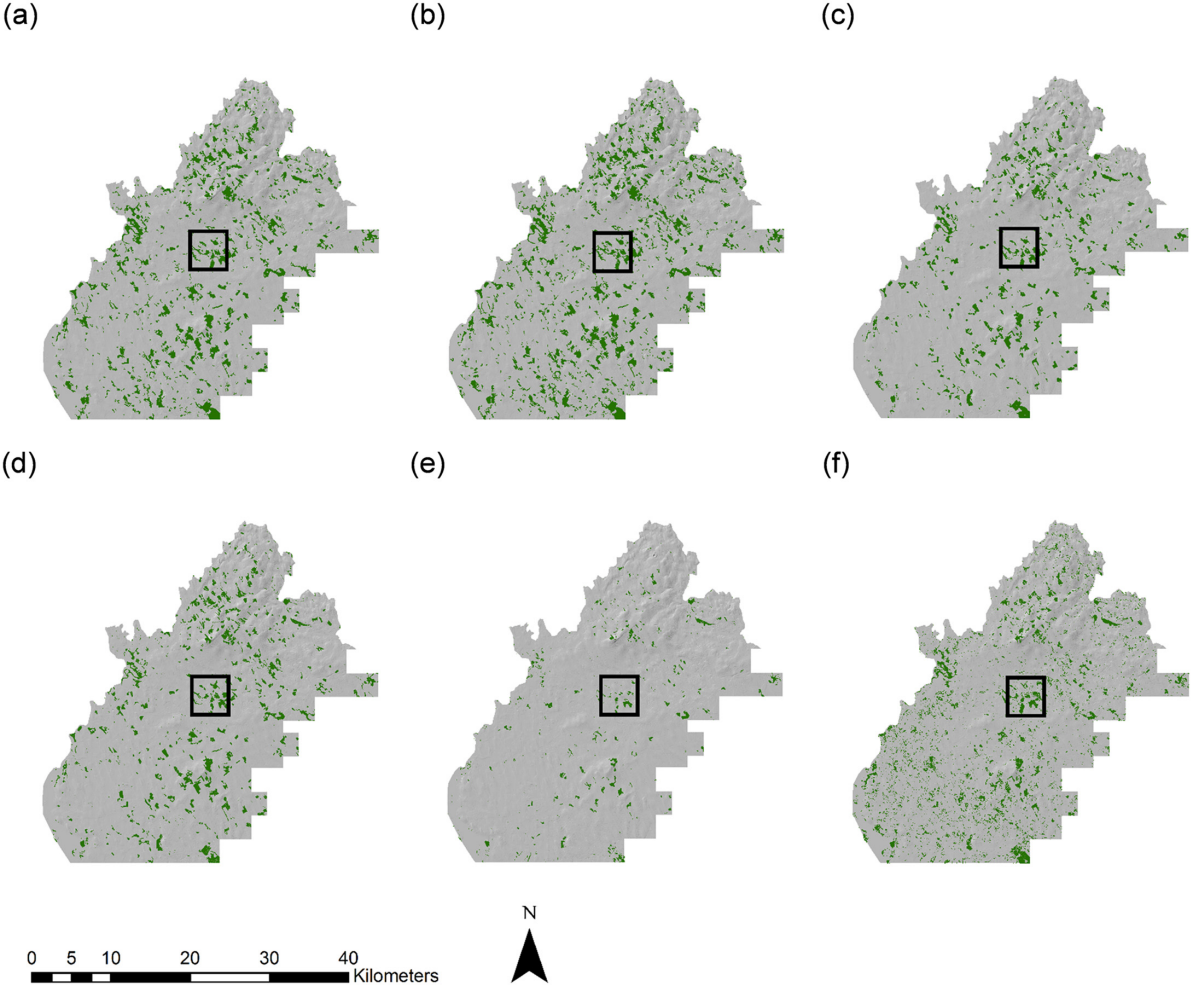
Extent of forest cover in Longford based on (a) SAR RF-5, (b) SAR ERT-5, (c) Forestry2010, (d) Prime2, (e) JRC Forest Map 2006, and (f) Global Forest Change map. The boxed area indicates the zoom-in area shown in Fig 6.

**Fig 6:**
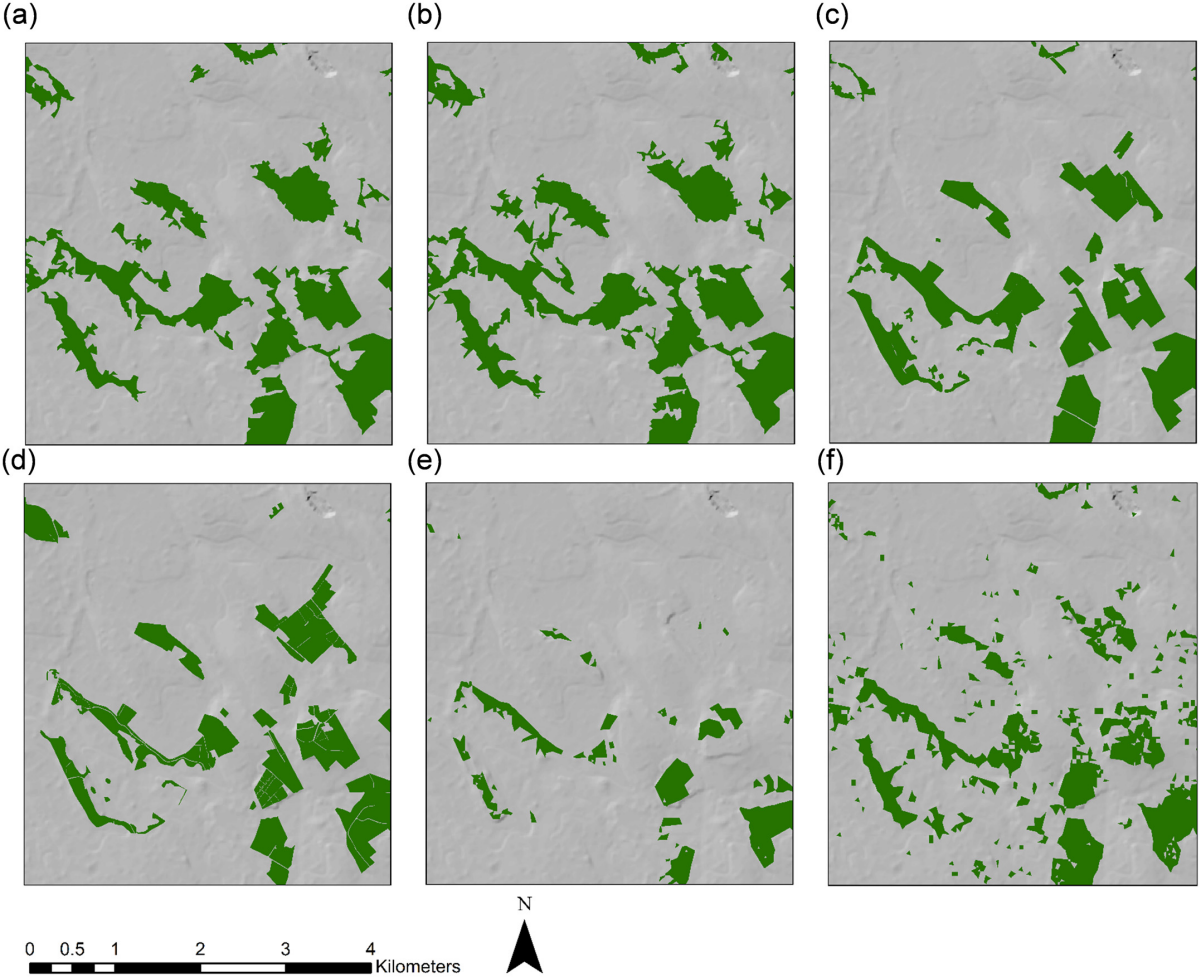
Zoomed-in (1: 60,000) extent of forest cover in Longford based on (a) SAR RF-5, (b) SAR ERT-5, (c) Forestry2010, (d) Prime2, (e) JRC Forest Map 2006, and (f) Global Forest Change map.

**Fig 7:**
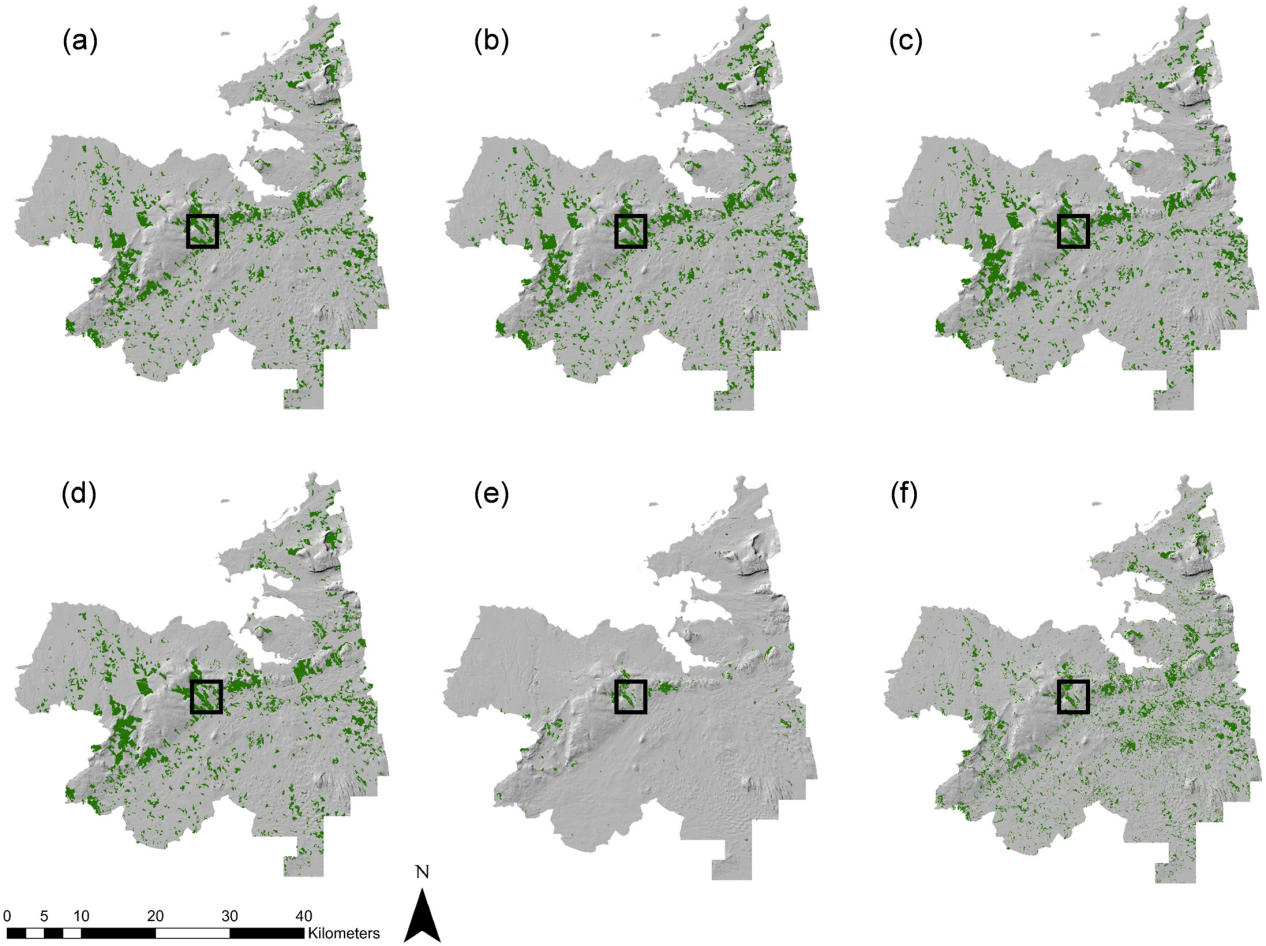
Extent of forest cover in Sligo based on (a) SAR RF-5, (b) SAR ERT-5, (c) Forestry2010, (d) Prime2, (e) JRC Forest Map 2006, and (f) Global Forest Change map. The boxed area indicates the zoom-in area shown in Fig 8.

**Fig 8:**
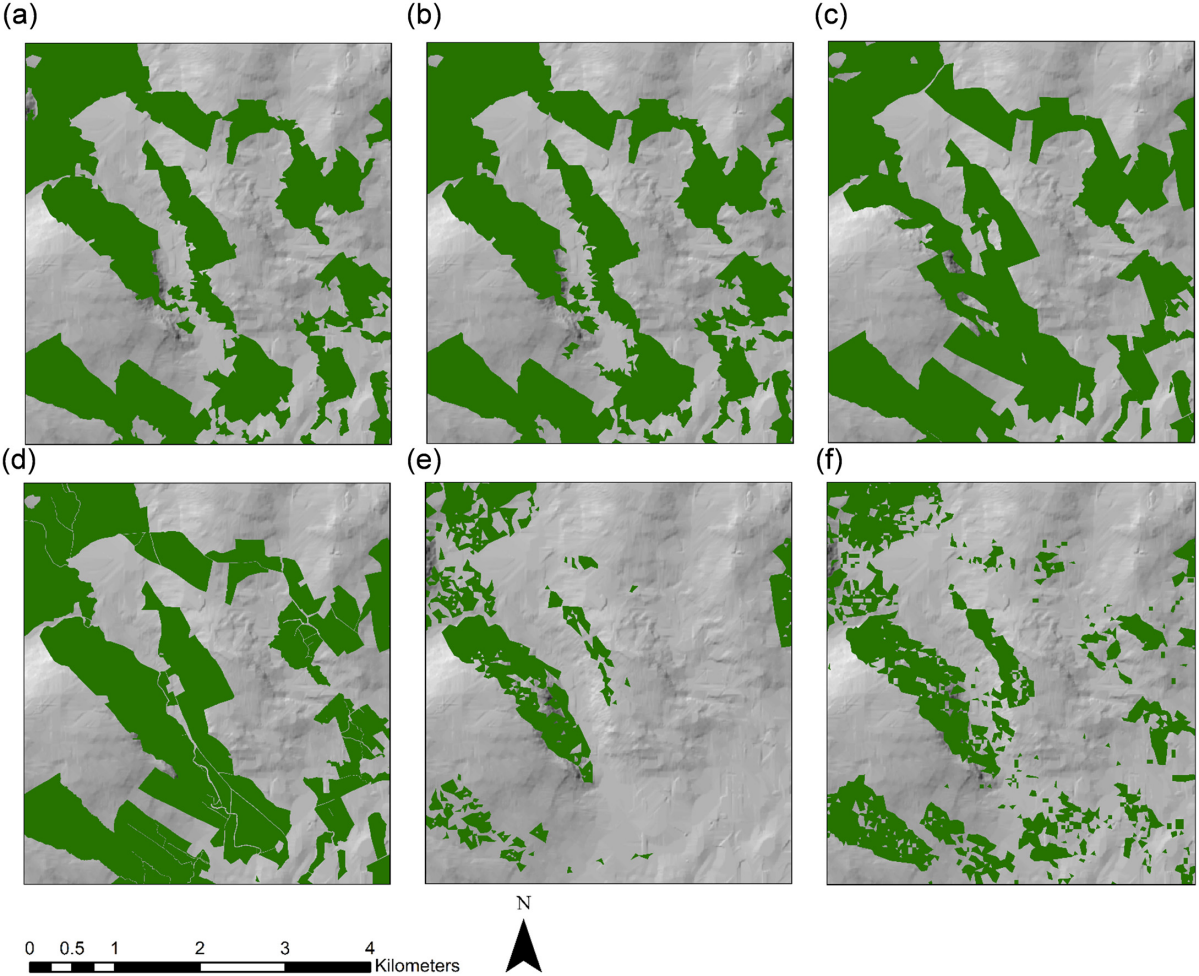
Zoomed-in (1: 60,000) extent of forest cover in Sligo based on (a) SAR RF-5, (b) SAR ERT-5, (c) Forestry2010, (d) Prime2, (e) JRC Forest Map 2006, and (f) Global Forest Change map.

A comparative analysis of forest area estimates for Longford and Sligo for the different forest cover datasets was carried out and is presented in Table 6. Considerable variation in the spatial extent of forest cover was found between the sources investigated (see Figs 5–8 and S3 and S4 Figs). In Longford, total forest area varied from 11,848.8 ha using SAR derived estimates, to 2153.1 ha using the JRC Forest Map 2006. Mean forest size was highest in SAR derived estimates (12.51 ha) and lowest in the GFC map (1 ha). In both counties, the GFC map indicated a much larger number of small, fragmented forest parcels than other datasets (Figs 6 and 8). In Sligo, total forest area ranged from 20,585.6 ha in the NFI estimate, to 2043.8 using the JRC Forest Map 2006. Mean forest size was highest in Forestry2010 (15.16 ha) and lowest in the GFC map (1.25 ha). In all cases, population of the Prime2 object-based spatial data storage model led to a reduction in the total estimated forest area. For example, following Prime2 population, the forest cover area estimation for Longford using SAR decreased from 11,848.8 ha to 9576.6 ha. The greatest proportionate reduction in area following Prime2 population was recorded in the GFC map, decreasing from 8368.5 ha to 5763.9 ha (~31%) in Longford, and from 16,559.2 ha to 11,972.8 ha (~28%) in Sligo. For Longford, the highest overall accuracy was recorded for the Forestry2010 dataset: 96.57%, increasing to 97.42% following Prime2 population. For Sligo non-Prime2 populated products, the highest overall accuracy was again recorded in the Forestry2010 dataset (95.52%). The highest overall accuracy was obtained for the Sligo Prime2 populated map (97.43%).

**Table 6:** Forest area (ha), mean forest size (ha), and accuracy (based on independent accuracy assessment dataset) for forest cover estimation in both non-Prime2 populated and Prime2 populated maps, in Longford and Sligo.

Region	Non Prime2 populated					Prime2 populated				
	Forest area (ha)	Mean forest size (ha)	Producer`s Accuracy (%)	User`s Accuracy (%)	Overall accuracy (%)	Forest area (ha)	Mean forest size (ha)	Producer`s Accuracy (%)	User`s Accuracy (%)	Overall accuracy (%)
**Longford**										
SAR* (RF)	10,086.70	13.35	92.21	91.03	96.28	8,184.90	3.87	90.91	93.33	96.57
SAR* (ERT)	11,848.80	12.51	94.81	89.02	96.28	9,346.60	3.64	92.21	91.03	96.28
Forestry2010	7,314.60	9.55	88.31	95.77	96.57	6,724.60	4.73	90.91	97.22	97.42
Prime2	-	-	-	-	-	6,923.70	3.93	87.01	97.1	96.57
NFI	6,769.90	-	-	-	-	-	-	-	-	-
JRC	2,153.10	2.26	12.99	90.91	80.57	1,870.30	3.94	16.88	92.86	81.42
GFC	8,368.50	1	71.43	85.94	91.14	5,763.90	2.78	68.83	91.38	91.71
**Sligo**										
SAR* (RF)	17,731.30	18.29	77.78	88.61	94.68	16,658	5.92	75.56	95.77	95.41
SAR* (ERT)	19,533.90	19.79	81.11	82.95	94.13	17,745.80	5.77	81.11	92.41	95.78
Forestry2010	17,827.20	15.16	77.78	92.11	95.52	17,036.00	7.14	75.56	93.15	95.05
Prime2	-	-	-	-	-	18,297.80	6.53	88.89	95.24	97.43
NFI	16,571.10	-	-	-	-	-	-	-	-	-
JRC	2,043.70	2.44	2.22	100	83.85	1,546.30	5.97	3.33	100	84.04
GFC	16,559.20	1.25	58.89	77.94	90.46	11,972.80	2.82	60	91.52	92.48

*The SAR forest area estimates for Longford and Sligo are derived from the RF-5 and ERT-5 products

## Discussion

### 4.1 Forest backscatter signatures

**Fig 9:**
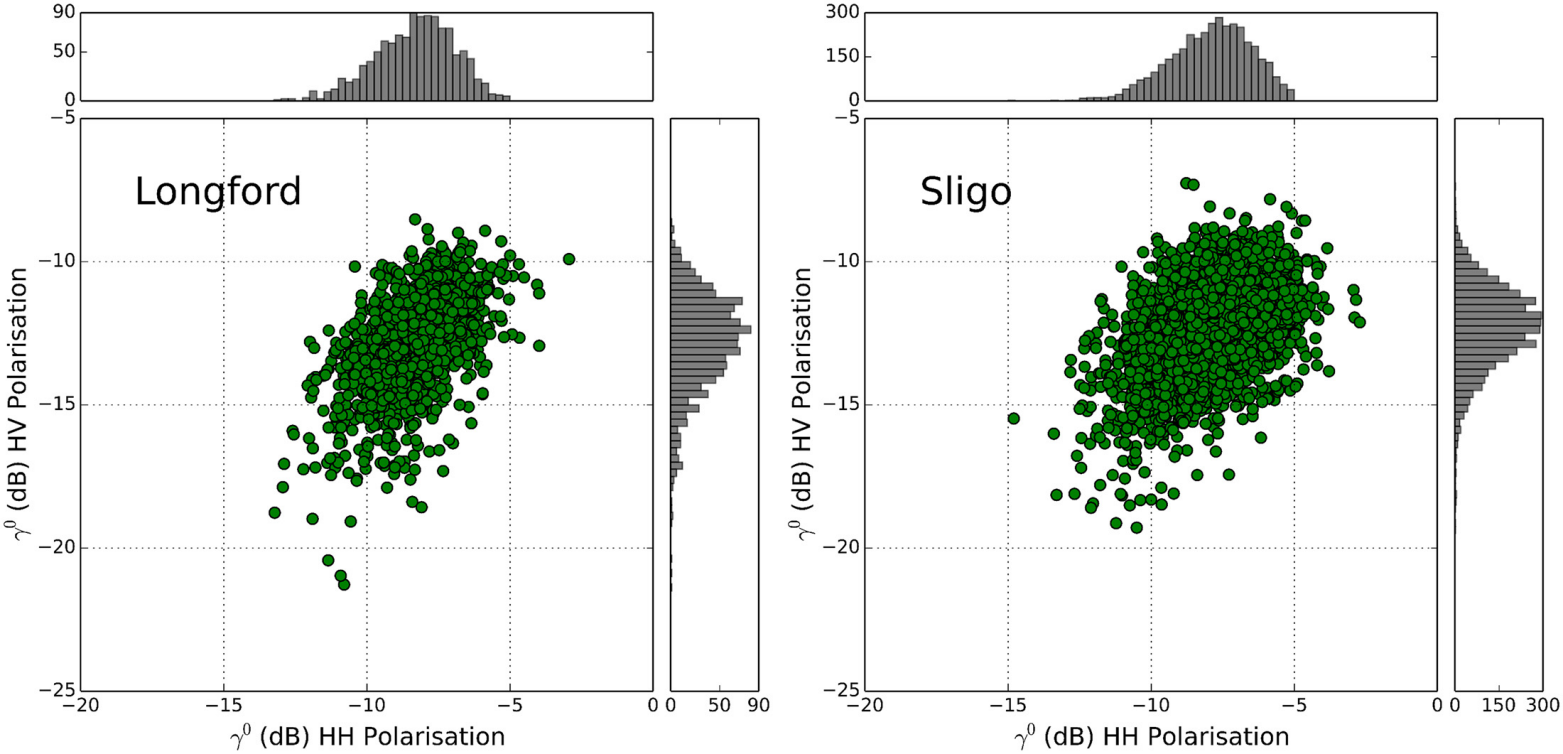
Comparison of HH and HV γ° backscatter for Forest training samples at Longford and Sligo.

The amount of radar backscatter received from a forest canopy depends upon the system frequency, polarisation and incidence angle, as well as canopy parameters such as structure and moisture. L-band signal penetrates deeper into the canopy than shorter wavelengths (e.g. X- and C- band) and interacts with the larger canopy components (e.g. large branches, trunk-ground interactions) rather than leaves or twigs. Fig 9 displays a comparison of HH and HV γ° backscatter values for forest training samples at Longford and Sligo. The HH backscatter was higher compared to the HV backscatter for both counties, with both the HH and HV backscatter being higher in Sligo than Longford. The diversity in backscatter responses may be attributed to the scattering processes associated with different forest conditions. Generally, dense mature canopies will result in an increase in HV and HH backscatter. Forest canopies usually depolarise the radar signal, resulting in a strong HV signal. However, strong HV backscatter (and increased depolarisation) may also be caused by forest detritus left after a disturbance event [40]. Such surface conditions would also lead to a strong HH signal, due to increased surface roughness and additional corner reflectors.

### 4.2 Comparison with accuracies for other land cover types

The principal focus of this study is the ability of radar to distinguish between forest and non-forest covers in a fragmented landscape. For a thorough investigation, the achieved classification results were also assessed in terms of the individual classes that make up the non-forest class (i.e. the 472 grassland, 71 crop, 296 peatland, 182 settlement and 168 water samples that make up the 1189 non-forest samples for Longford, and the 502 grassland, 37 crop, 1132 peatland, 1293 settlement, 685 water and 296 exposed rock samples that make up the 3945 non-forest samples for Sligo). In addition, these accuracies were compared with an additional non-parametric (Support Vector Machines—SVM) and parametric classifier (Maximum Likelihood—ML) (Table 7). The ERT classifier outperforms the RF classifier for both counties, although only marginally in Sligo. The SVM also performs well and the lowest accuracies are obtained by using the traditional ML classifier. Although these other land covers were not of primary interest in this study, the results outline the potential of the adopted approach for mapping different cover types, as for example, has been shown by Barrett et al., [14] for distinguishing between grassland types.

**Table 7:** Longford and Sligo classification results for multiple classes (RF = Random Forests, ERT = Extremely Randomised Trees, SVM = Support Vector Machines, ML = Maximum Likelihood, PA = Producer’s Accuracy, UA = User’s Accuracy).

	Longford									Sligo								
	RF		ERT		SVM		ML			RF		ERT		SVM		ML		
	PA	UA	PA	UA	PA	UA	PA	UA	# Samples	PA	UA	PA	UA	PA	UA	PA	UA	# Samples
Forest	0.97	0.99	0.97	1.00	0.96	0.98	0.96	0.94	1189	0.98	0.99	0.98	0.99	0.97	0.98	0.95	0.96	3945
Grassland	0.88	0.94	0.90	0.95	0.88	0.89	0.83	0.84	472	0.86	0.79	0.88	0.79	0.77	0.73	0.46	0.40	502
Water	1.00	1.00	1.00	1.00	1.00	1.00	0.99	0.98	168	0.97	0.98	0.96	0.98	0.93	0.96	0.72	0.94	685
Settlement	0.91	0.76	0.98	0.81	0.89	0.74	0.61	0.65	182	0.93	0.91	0.94	0.91	0.87	0.87	0.83	0.68	1293
Peatland	0.89	0.99	0.92	0.99	0.89	0.97	0.77	0.93	296	0.94	0.98	0.94	0.98	0.93	0.96	0.80	0.87	1132
Crop	0.87	0.18	0.83	0.34	0.53	0.35	0.04	0.01	71	1.00	0.32	0.95	0.51	0.71	0.68	0.28	0.14	37
Exposed Rock	/	/	/	/	/	/	/	/	-	0.91	0.90	0.89	0.88	0.80	0.70	0.39	0.35	296
OA	93.9%		95.1%		92.6%		86.9%		/	95.7%		95.9%		92.8%		83.7%		/
Kappa	0.91		0.93		0.89		0.81		/	0.94		0.94		0.90		0.76		/

### 4.3 Comparison of SAR-derived forest cover maps with existing forest cover estimates

Observed differences between SAR-derived maps, Forestry2010, and NFI are of particular importance as these datasets are commonly used in national reporting statistics. For Longford, the lowest SAR-derived estimate was approximately 40% greater than Forestry2010 and NFI estimates. This discrepancy may be partly explained by changing land-cover trends. Longford has a high proportion of raised bog land-cover, much of which has been used for industrial harvesting of peat for electricity production and domestic heating [83]. Anecdotal evidence suggests that, due to changing socio-economic circumstances and environmental concerns, industrial practices on many of these peatlands have recently been abandoned leading to the development of successional woodlands. Within the Forestry2010 dataset, recent, naturally developing forests are not accounted for and hence may be underrepresented. Conversely, SAR-derived maps may have incorrectly identified non-forest scrub vegetation (such as *Ulex* spp.) as forest, leading to an over-estimation of the total forest area. These areas support a lower L-band backscatter (see Fig 9) and could be mapped as a separate class [84].

To our knowledge, this is one of the first studies worldwide to assess the usefulness of the GFC map for forest monitoring on a regional scale, whereby accuracies are compared to existing and novel national forest cover datasets. Although GFC-derived forest area estimates were within 25% of national (Forestry2010 and NFI) and SAR-derived estimates, overall accuracy was consistently lower in GFC maps than other sources (with the exception of FMAP2006). Visual inspection of forest maps indicates a higher number of small forest parcels in the GFC dataset (Figs 6 and 8). The possible erroneous inclusion of non-forest areas of tree cover as forest in the GFC dataset may have occurred due to difficulties with harmonising forest definitions between the different estimation methodologies. The Irish national forest definition [85] was applied for this study and attempts were made to align all datasets to this definition. Nevertheless, the original GFC dataset was designed to capture percentage cover of all vegetation >5 m in height, rather than forest as described by the national definition. Although forest cover in Ireland is low compared to other European countries [1], a large area of trees outside forests in the form of hedgerows and scrub exists [30]. The GFC dataset may have incorrectly incorporated some of these areas as forest. It is important to note that no post-classification filtering was carried out on JRC or GFC forests maps. This process would be unlikely to improve accuracies of the JRC map due to its large under-representation of forest cover but may influence accuracies of GFC forest maps. Global and pan-continental forest cover maps such as those investigated in this study are essential instruments in the long-term monitoring of large-scale land cover changes. However, the reduced accuracies of these datasets in comparison to national and the SAR-derived forest maps, indicate that caution should be exercised when applying these datasets to nuanced local forest conditions, such as highly sparse and fragmented forest landscapes. Currently, particularly in developed counties where pre-existing forest cover estimation methodologies have been optimally developed for local circumstances, their use for national reporting may be limited.

In almost all cases, population of the object-based Prime2 storage model with other forest cover maps resulted in increased overall accuracy of maps. Prime2 is a highly detailed spatial framework of all topological features on the Irish landscape. Rather than being pixel-based, it has the advantage of incorporating landscape features and real-world objects. While increases in overall accuracy between Prime2 populated and non-Prime2 populated maps are small, this trend supports the potential use of Prime2 as a spatial framework for determining forest (and general land) cover in Ireland using EO data. A similar product, the Ordnance Survey UK MasterMap, has been used in conjunction with optical imagery to produce a land-cover map for the United Kingdom with an overall accuracy of 83% based on 9127 field validation points [86].

As identified in previous comparative studies (e.g. [17,78]), limitations exist with regard to the spatial and temporal harmonisation of datasets. In some cases, differences in basic methodologies meant that spatial and temporal alignment of all datasets was not possible. As the NFI is a sample based estimation methodology, accuracy comparison with other estimates was not possible. Similarly, high spatial resolution of training data within the FMAP 2006 product may have led to an underrepresentation of forest cover in our study areas. The FMAP 2006 has reported overall classification accuracy on a pan-European level of 88.0%. However, for countries such as Ireland which have a low proportion of forest, there is a strong under-estimation of forest cover (see Table 6 and [11]). This is due to the minimum mapping unit of 25 ha of the CLC2006 which was used to train the classifier. As the mean forest size in Ireland is < 25 ha, many forest areas were not included in the training dataset used to produce the FMAP 2006. Similar difficulties in reporting forest-related land-cover changes in using CORINE data were noted by Black *et*. *al*. [87]. Temporal differences in data capture will also have contributed some variation in the estimates provided. Data acquisition for the FMAP 2006 was carried out between 2005 and 2007 and hence, recently afforested areas would have been omitted from this dataset, although this is unlikely to explain the large under-representation of the forest area by this product.

## Conclusions

In countries with near constant cloud-cover, radar EO data has the potential to produce high-accuracy, high-resolution forest cover maps. Of particular note is the possible use of SAR data in Land-Use, Land-Use Change and Forestry (LULUCF) sector of UNFCCC inventory reports, whereby annual updates of forest area, afforestation and deforestation areas are required. SAR-derived forest cover maps for two Irish regions displayed high accuracies following independent accuracy assessment and were comparable with datasets currently used in national forest reporting. These findings indicate that dual-polarisation radar could aid forest inventories. However, SAR forest mapping is primarily a *land-cover* mapping technique, whereas LULUCF reporting is based on *land-use*. In countries where the principal silvicultural method is clear-felling followed by replanting, a change in forest cover may be not associated with a change in land-use. Thus, for reporting land-use change events, SAR-based forest cover change identification should be used in combination with data from other sources such as ground surveys, ancillary data and other sensors (e.g. optical) [21]. SAR-derived maps of tree cover may also be applicable to the assessment of “trees outside the forest” [88] which are often overlooked in standard inventory methodologies such as NFIs [89].

Although ALOS PALSAR is no longer operational, a follow-on mission, ALOS-2 [90] was successfully launched on 24^th^ May 2014. Furthermore, continuity of observations will be facilitated by the ever-increasing number of radar sensors being launched. This, in addition to free and open access policies for certain sensor data (e.g. Sentinel-1A/B—launched 3^rd^ April 2014) further constitute a basis for incorporating SAR-derived forest cover estimates into national reporting mechanisms. The reduced accuracies of global and pan-continental forest cover maps investigated in this study indicate that caution should be exercised when applying these datasets to nuanced local forest conditions.

## Supporting Information

S1 Fig Location of classification training samples for Longford. Green dots refer to Forest samples and white dots depict Non-Forest samples.
(TIF)

S2 Fig Location of classification training samples for Sligo. Green dots refer to Forest samples and white dots depict Non-Forest samples.
(TIF)

S3 Fig Zoomed-in (1: 150,000) extent of forest cover in Longford based on (a) SAR RF5, (b) SAR ERT5, (c) Forestry2010, (d) Prime2, (e) JRC Forest Map 2006, and (f) Global Forest Change map. (TIF)

S4 Fig Zoomed-in (1: 150,000) extent of forest cover in Sligo based on (a) SAR RF5, (b) SAR ERT5, (c) Forestry2010, (d) Prime2, (e) JRC Forest Map 2006, and (f) Global Forest Change map. (TIF)

S1 Table Longford and Sligo classification results for multiple classes (RF = Random Forests, ERT = Extremely Randomised Trees, SVM = Support Vector Machines, ML = Maximum Likelihood, PA = Producer’s Accuracy, UA = User’s Accuracy) without ancillary soil data (XLSX)

S2 Table Longford and Sligo classification results for multiple classes (RF = Random Forests, ERT = Extremely Randomised Trees, SVM = Support Vector Machines, ML = Maximum Likelihood, PA = Producer’s Accuracy, UA = User’s Accuracy) without ancillary elevation data (XLSX)

S3 Table Longford and Sligo classification results for multiple classes (RF = Random Forests, ERT = Extremely Randomised Trees, SVM = Support Vector Machines, ML = Maximum Likelihood, PA = Producer’s Accuracy, UA = User’s Accuracy) without ancillary soil and elevation data (XLSX)
