# Supplementary figures and images for: Forest Cover Estimation in Ireland Using Radar Remote Sensing: A Comparative Analysis of Forest Cover Assessment Methodologies
Source: PLoS One. 2015 Aug 11;10(8):e0133583. doi: 10.1371/journal.pone.0133583 (PMC4532497; doi:10.1371/journal.pone.0133583)

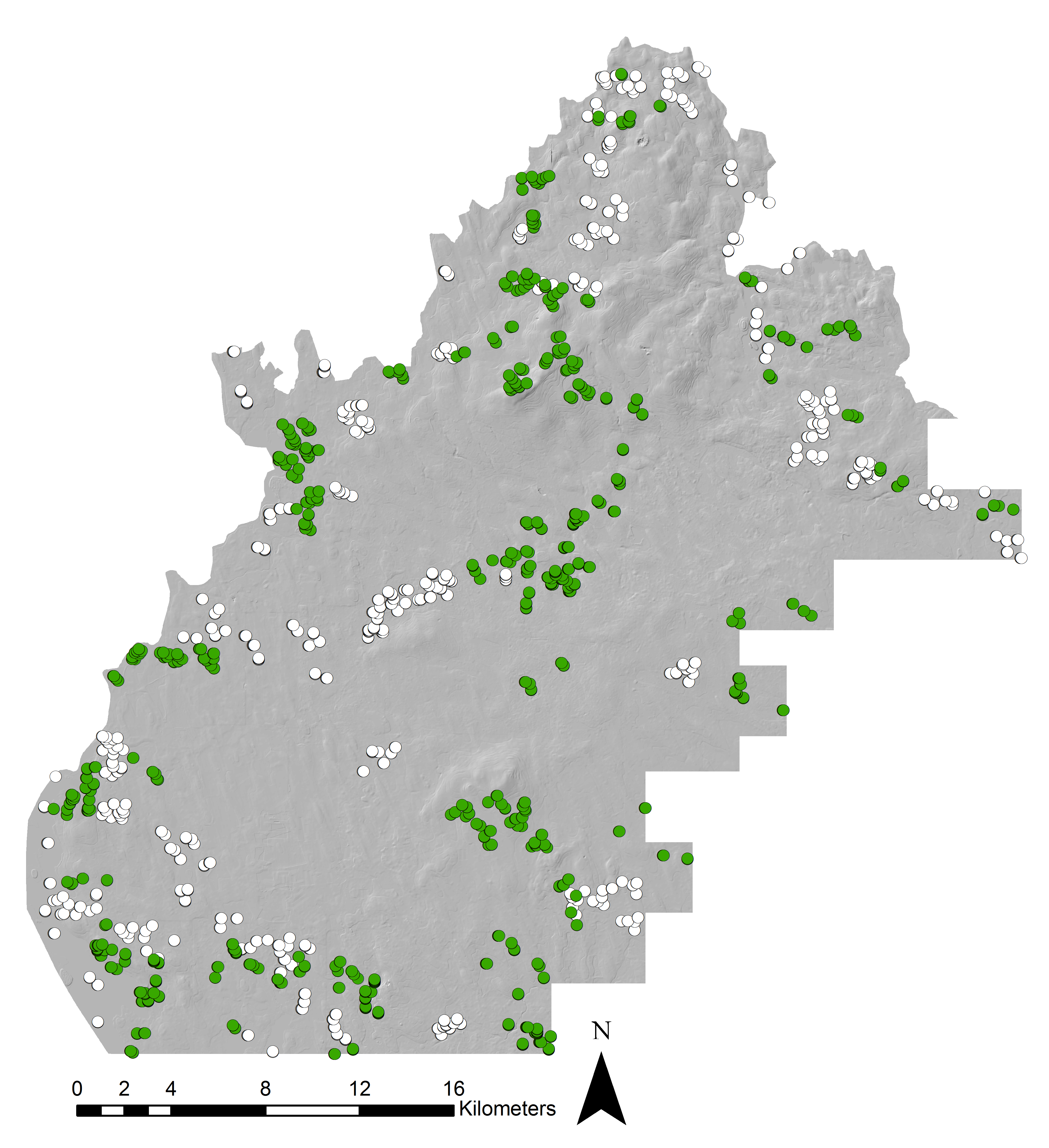

Supplement: S1 Fig — Green dots refer to Forest samples and white dots depict Non-Forest samples. (TIF) [file pone.0133583.s001.tif]

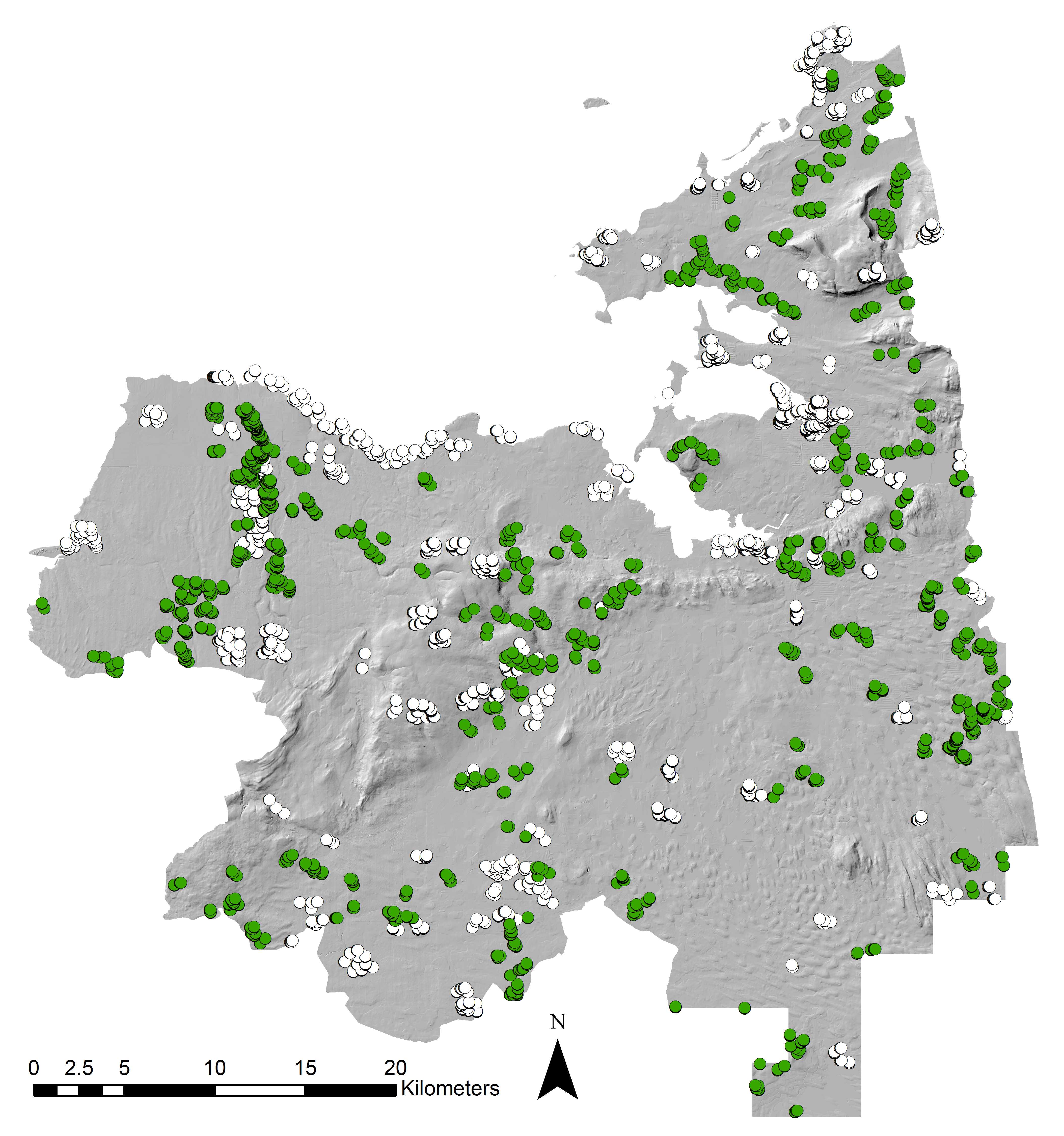

Supplement: S2 Fig — Green dots refer to Forest samples and white dots depict Non-Forest samples. (TIF) [file pone.0133583.s002.tif]

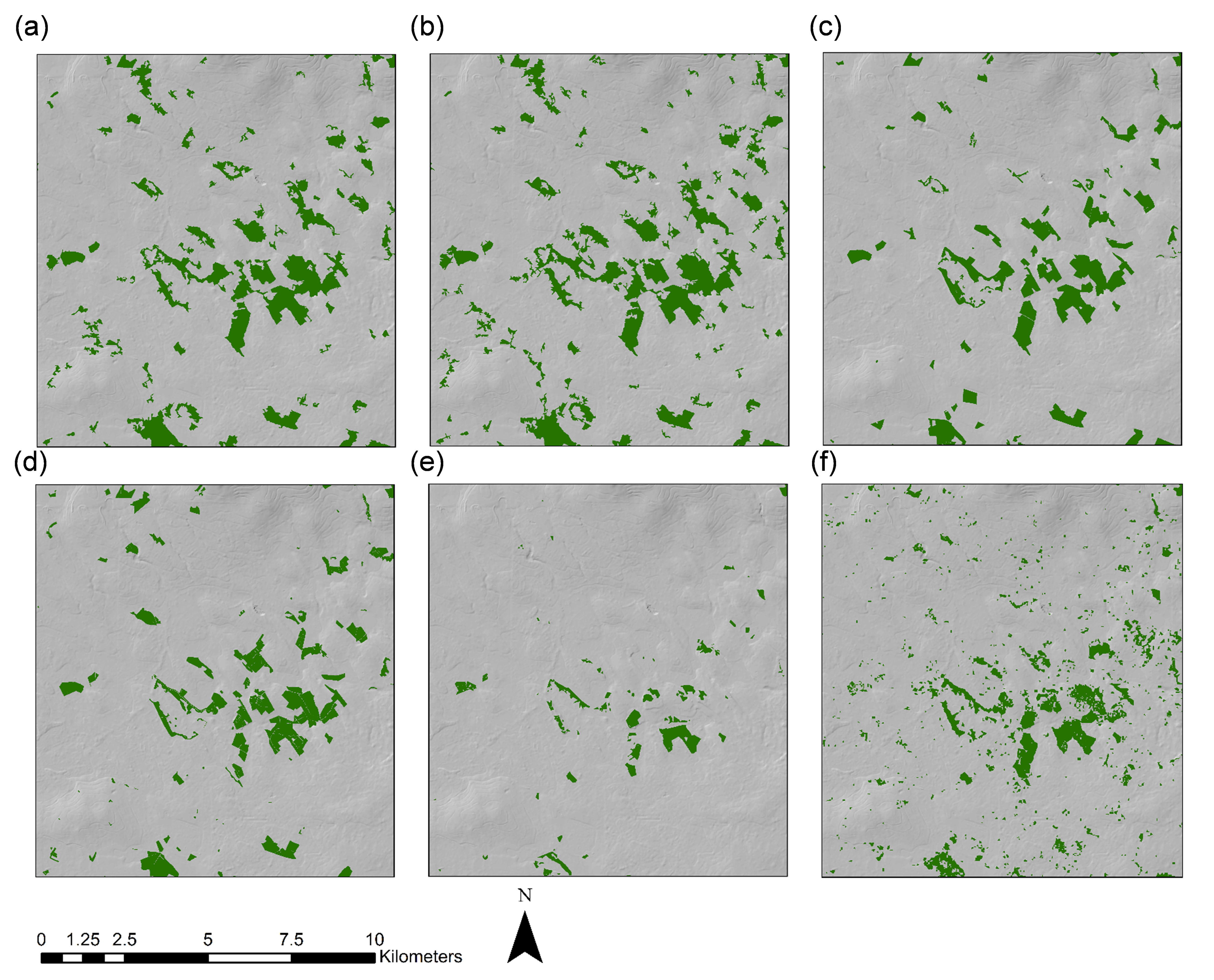

Supplement: S3 Fig — (TIF) [file pone.0133583.s003.tif]

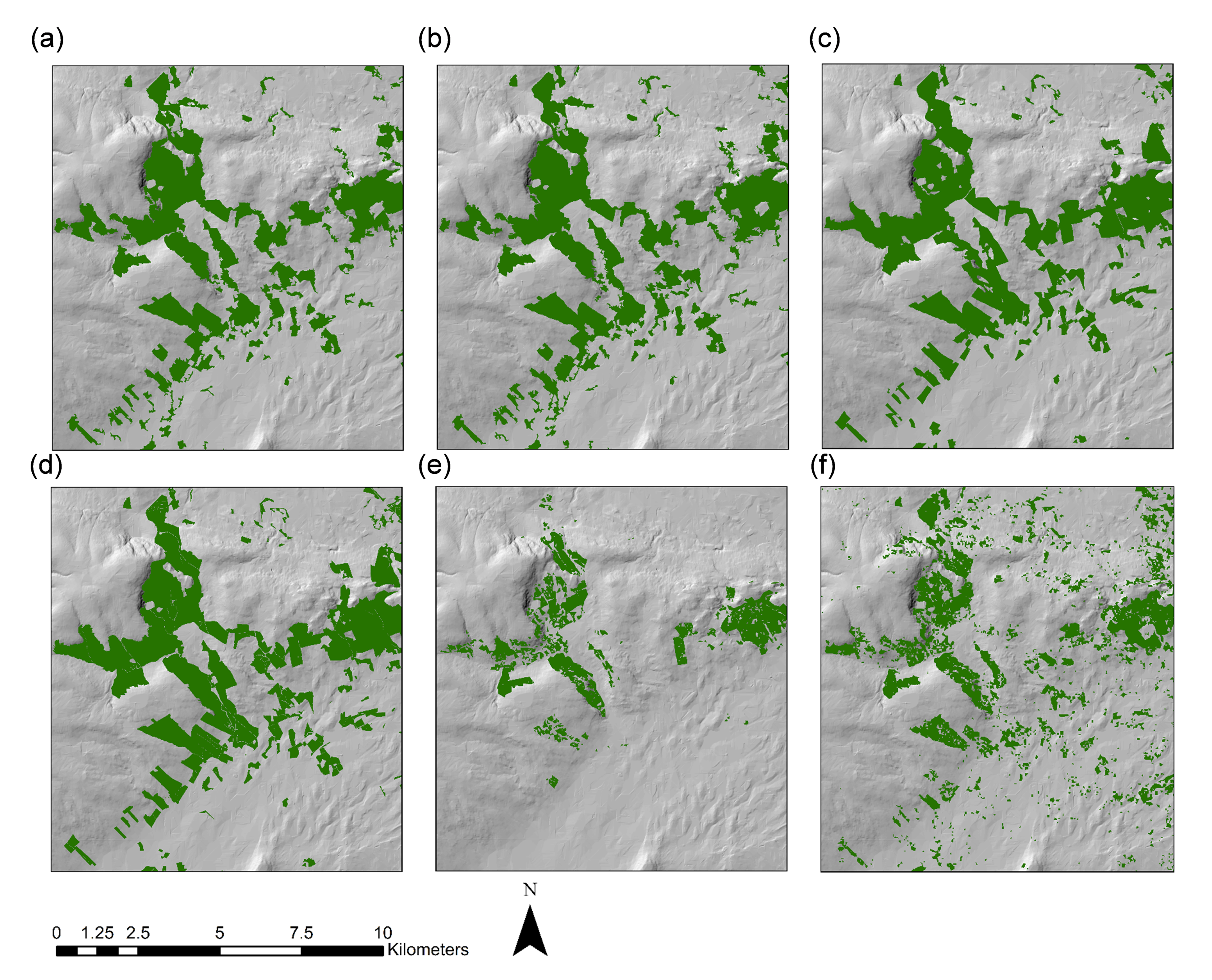

Supplement: S4 Fig — (TIF) [file pone.0133583.s004.tif]
